# An observational study on cough in children: epidemiology, impact on quality of sleep and treatment outcome

**DOI:** 10.1186/1745-9974-8-1

**Published:** 2012-01-23

**Authors:** Francesco De Blasio, Peter V Dicpinigaitis, Bruce K Rubin, Gianluca De Danieli, Luigi Lanata, Alessando Zanasi

**Affiliations:** 1 Respiratory Medicine and Pulmonary Rehabilitation Section, Clinic Center, Private Hospital, Naples, Italy; 2 Department of Medicine, Albert Einstein College of Medicine and Montefiore Medical Center, Bronx, NY, USA; 3 Department of Pediatrics Virginia Commonwealth University Physician in Chief, Children's Hospital of Richmond, USA; 4 Medical Department, Dompé spa, Milan, Italy; 5 Pneumology Unit, University of Bologna, S.Orsola Malpighi Hospital, Bologna, Italy

**Keywords:** Cough, Antitussive, Children, Levodropropizine, Cloperastine, Codeine

## Abstract

**Background:**

Cough is one of the most frequent symptoms in children and is the most common symptom for which children visit a health care provider.

**Methods:**

This is an observational study on acute cough associated with upper respiratory tract infection (URTI) in children. The study evaluates the epidemiology and impact of cough on quality of sleep and children's activities, and the outcome of cough with antitussive treatments in pediatric routine clinical practice. Study assessments were performed through a pediatric cough questionnaire (PCQ), developed by the Italian Society of Cough Study. A total of 433 children visited by family care pediatricians for acute cough due to a URTI were enrolled in this study, with mean age of 6.1 years (SD 3.6). Cough type, duration, severity and frequency, cough impact on sleep disturbances of children and parents and on school and sport activities were assessed at baseline. In a subset of 241 children who were either treated with antitussive drugs (levodropropizine n = 101, central antitussives n = 60) or received no treatment (n = 80), the outcome of cough after 6 days was analyzed in terms of resolution, improvement, no change, or worsening. Descriptive analysis, χ^2 ^test, and multivariate analysis with stepwise logistic regression were performed.

**Results:**

Cough disturbed sleep in 88% of children and 72% of parents. In children treated with cough suppressants, the duration, type, intensity, and frequency cough were similar at baseline in the two groups respectively treated with levodropropizine and central antitussives (cloperastine and codeine). Both levodropropizine and central drugs reduced cough intensity and frequency. However, percentage of cough resolution was higher with levodropropizine than with central antitussives (47% vs. 28% respectively, p = 0.0012).

**Conclusions:**

Acute cough disturbs sleep in most children and their parents. Both levodropropizine and central antitussives reduced cough intensity, with levodropropizine producing a higher cough resolution rate.

## Background

Cough is one of the most frequent symptoms in children [1,2] and is one of the most common reasons for which parents seek medical attention for their child [3]. In most children, acute cough is due to viral upper respiratory tract infection (URTI), i.e., the common cold [4]. It is recognized that preschool and school children might suffer from acute respiratory infections 6 to 8 times a school year and can cough 140 coughs daily with a URTI [5].

Cough resulting from URTI may be a distressing symptom, and empiric treatment with antitussive agents is often used [6]. Among antitussive drugs available for the treatment of cough in children, codeine and cloperastine are centrally acting agents (opioids and non opioids) that are believed to inhibit cough primarily by their effect on the cough center, while levodropropizine is a non-opioid agent whose suggested peripheral antitussive action may result from inhibition of the cough reflex at the peripheral nerve level (sensory C-fibres) [7] with possible modulation of sensory neuropeptide levels within the respiratory tract [8].

The aim of this study was to evaluate the epidemiology of acute cough associated with URTI in children, the impact of acute cough on quality of sleep of children and their parents, the impact of acute cough on children's sport and school activities, and acute cough outcome with antitussive treatments.

## Methods

This is an observational study on pediatricians' routine clinical practice, including all children who presented to the offices of four family care pediatricians due to acute cough (i.e., onset ≤ 3 weeks) associated with a URTI from 1st February 2010 to 30th April 2010. Study assessments were performed through a specific pediatric cough questionnaire (PCQ), developed and approved by the Scientific Committee of the Italian Society of Cough Study (see Appendix). Baseline assessment was performed during the first study visit by the pediatrician, who interviewed parents and/or patients and compiled the first part of the PCQ, addressing the evaluation of type, duration, frequency and severity of cough, sleep disturbances of both children and parents (quality of sleep), and impact of cough on school activities and sports/games. Pediatricians were free to prescribe the most appropriate treatments for cough based on their clinical practice experience. The PCQ was given to the children's parents for self assessment to be performed after 6 days from the first study visit, compiling the second part of the questionnaire reporting actually administered treatments, and the outcome of cough in terms of resolution, improvement, no change, or worsening. Patients were revisited after one week from the first visit, and questionnaires were collected by the physician, as well as any adverse events experienced by the child.

### Statistics

Continuous data are presented as mean +/- standard deviation. Categorical and discrete data are presented as frequency and percentages. Differences between groups were tested using the χ^2 ^test for categorical and discrete variables. A multivariate analysis was performed. Using a stepwise logistic regression we assessed how changes in cough severity related to the age of children, the presence of concomitant respiratory diseases, the type of cough, the use of antibiotics, and the antitussive therapy used.

## Results

### Epidemiology of Cough

**Figure 1:**
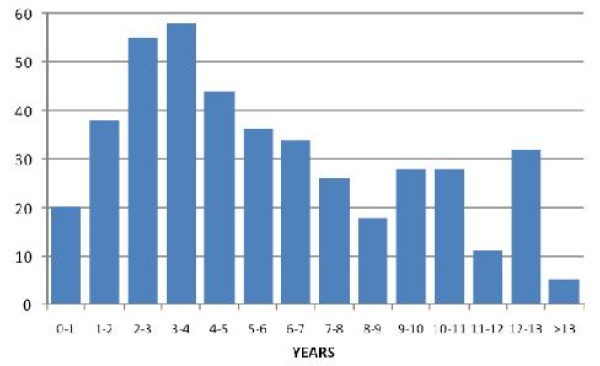
**Age of enrolled children**.

The total number of children enrolled in this study was 433. The mean age of children was 6.1 years (SD 3.6 - median 5.2 years). The youngest enrolled was 1 month, while the oldest was 14 years (Figure 1).

### Clinical findings

Clinical data including type, duration, frequency and severity of cough were recorded on the PCQ at the first study visit. In 88 (20.3%) patients a concomitant illness was found. Allergies were the most frequent conditions with an incidence of 9% (Table 1).

**Table 1:** Concomitant Respiratory Illnesses

**Concomitant respiratory illnesses**	20.3%
Allergies	9.0%
Asthma	6.2%
Bronchitis	3.0%
Rhinitis/Sinusitis	1.6%
Tonsillitis	0.5%

Frequency and intensity of cough were mostly reported as frequent and moderate, respectively (Table 2).

**Table 2:** Intensity and Frequency of cough

Intensity of Cough	Total
*Mild*	*81 (18.7%)*
*Moderate*	*237 (54.7%)*
*Severe*	*115 (26.6%)*
**Total**	**433 (100%)**

**Frequency of Cough**	**Total**

*Occasional*	*126 (29.1%)*
*Frequent*	*258 (59.6%)*
*Continuous*	*49 (11.3%)*
**Total**	**433 (100%)**

### Quality of sleep, sport and school activities

**Figure 2:**
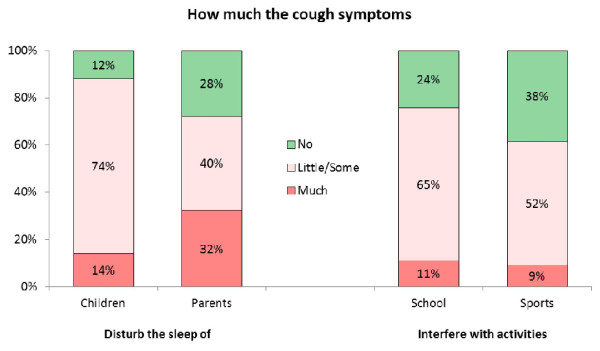
**Quality of sleep, sport and school activities**.

The PCQ evaluated the impact of cough on quality of sleep in children and their parents through four questions (Figure 2):

To what extent did cough symptoms disturb children's sleep?

To what extent did children's cough symptoms disturb their parent's sleep?

To what extent did children's cough symptoms interfere in their scholastic activities?

To what extent did children's cough symptom interfere with sport activities?

Regarding pre-scholar children and infants, scholastic activities were to be intended as activities at kindergarten/nursery/crèche, while sport activities were to be intended as games.

### Treatment of Cough

Of the total 433 patients, 80 received no treatment for cough since the doctor did not prescribe any medication, while 161 were treated with cough suppressant agents, which were the main drugs used in this study. Of these, 101 children received levodropropizine and 60 patients were treated with central antitussives (51 with cloperastine, and 9 with codeine).

The outcome of cough after 6 days was analyzed in terms of resolution, improvement, no change, or worsening in this subset of 241 children who were treated with antitussive drugs or received no treatment.

Regarding patients' characteristics at baseline (Table 3), the two groups respectively treated with levodropropizine and central antitussives (cloperastine and codeine) were similar for cough duration (number of days since cough onset), cough type, intensity, and frequency. Average age of patients treated with central antitussives was approximately 1 year and a half higher than patients who received levodropropizine, and this appears to be consistent with current routine clinical practice in the pediatric population, considering that codeine is contraindicated in children below 2 years. As expected in a real life observational study in which therapeutic decisions were driven by physicians' opinions based on their clinical practice experience, cough intensity and frequency were significantly lower in the group of patients for which pediatricians decided not to prescribe any treatment than in the two groups treated with levodropropizine and central antitussives, respectively.

**Table 3:** Patients Characteristics at Baseline

	Levodropropizine (LDP) n = 101	Central Antitussives (CA) n = 60	No Treatment (NT) n = 80	p * p < 0,05 LDP. vs CA
**Age (years)**, mean (min-max)	5.9* (0.8-14.8)	7.3 (1.6-13.3)	6.3 (0.3-14.0)	NS LDP vs NT NS CA vs NT
**Cough Duration (days)**, mean (min-max)	4.6 (2-15)	5.8 (2-21)	4.7 (2-17)	NS
**Cough Type (%)**				
*Productive*	36.6%	25.0%	46.3%	NS
*Dry*	50.5%	63.3%	40.0%	NS
*Mix*	12.9%	11.7%	13.8%	NS
**Cough Intensity (%)**				
*Mild*	7.9%	8.3%	42.5%*	*p < 0.01 NT vs LDP/CA
*Moderate*	58.4%	53.3%	51.3%	NS
*Severe*	33.7%	38.3%	6.3%*	*p < 0.01 NT vs LDP/CA
**Cough Frequency (%)**				
*Occasional*	18.8%	10.0%	60.0%	NS
*Frequent*	65.3%	75.0%	36.3%	NS
*Continuous*	15.8%	15.0%	3.8%*	*p < 0.01 NT vs LDP/CA

### Correlation between treatment outcome and type of treatment received

**Figure 3:**
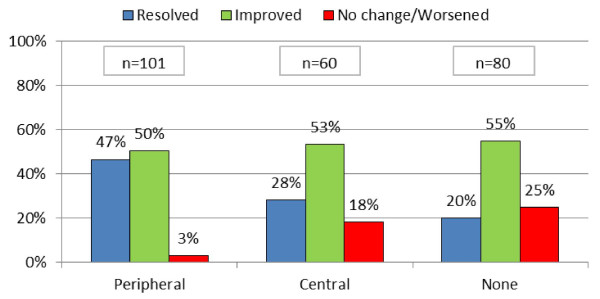
**Treatments outcomes**. Chi sq. (2 DF) = 13,4121, p = 0,0012

There was a significant difference in treatment outcomes between the groups receiving levodropropizine and those receiving central antitussives. The percentage of children reporting cough resolution was significantly higher with levodropropizine than with central antitussives (47% vs. 28% respectively, p = 0.0012), and no change/worsening was reported in 3% receiving levodropropizine vs. 18% for central antitussives (Figure 3). Twenty per cent of patients receiving no therapy reported resolution of cough, while 55% reported improvement of their symptoms.

### Correlation between intensity of cough and treatment outcomes

#### Moderate cough

**Figure 4:**
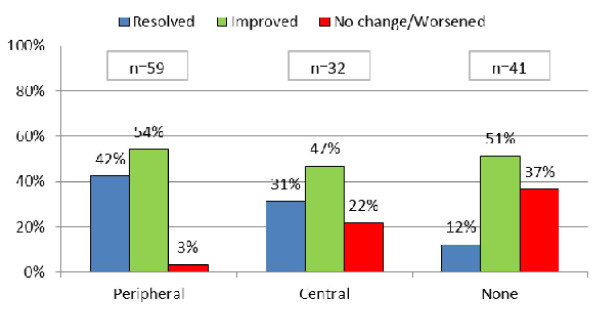
**Treatments outcomes - Moderate cough**.

Levodropropizine showed better treatment results than central antitussives and no therapy. A statistically significant difference was found (p < 0.05) in terms of percentage of cough resolution. Only 3% of patients in the levodropropizine group reported no change or worsening of cough symptoms vs. 22% and 37% for central antitussives and no therapy, respectively (Figure 4).

#### Severe Cough

**Figure 5:**
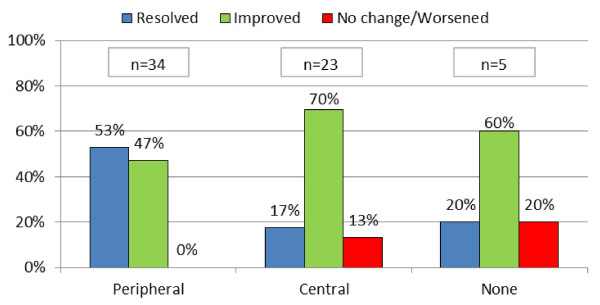
**Treatments outcomes - Severe cough**.

No patients who received levodropropizine reported no change/worsened cough (Figure 5).

#### Multivariate Analysis (Step-wise Logistic Regression)

A multivariate analysis by stepwise logistic regression was done to evaluate the probability of cough improvement related to clinical characteristics. The independent variable was resolution or improvement of cough while covariates were: age (continuous - months), concomitant diseases (Yes vs. No), type of cough (productive, dry, or mixed), association with antibiotics (Yes vs. No), antitussive therapy (levodropropizine, central antitussive, or none). The coefficients values derived from the logistic regression are shown in Table 4.

**Table 4:** Logistic analysis

COVARIATES	Contrast	**Coeff**.	SE	p
Age (months)	Continuous	-0.003	0.0033	0.311
Concomitant diseases Yes	vs. No	0.584	0.4020	0.147
Productive cough	vs. dry	-0.144	0.3200	0.652
	vs. mixed	-1.037	0.4010	*0.010
Association with antibiotics Yes	vs. No	0.471	0.3440	0.172
Levodropropizine	vs. Central antitussives	-2.026	0.6820	*0.003
	vs. No Treatment	-2.181	0.6750	*0.001

Cough resolution or improvement was most strongly associated with the type of antitussive used and type of cough. Children with productive cough tended to respond better than patients with mixed cough. Cough outcome was better with levodropropizine than with central antitussives. No statistically significant correlation was found with age, concomitant diseases or use of antibiotics.

## Discussion

Of the 433 children enrolled in this observational study, 52% were preschool ( < 6 years). The mean age of children was 6.2 years. In 88% of patients, acute cough due to URTI disturbed the children's quality of sleep, and the scholastic and sport activities were affected in 76% and 61% of children, respectively. The quality of parent's sleep was affected in 72% in parents

In the subset of 241 children who were treated with antitussive drugs (levodropropizine or central antitussives, n = 161) or received no treatment (n = 80), 80% were reported to have moderate or severe cough. More children were treated with levodropropizine (n = 101) than with central cough suppressants (codeine and cloperastine, n = 60). All antitussive drugs reduced cough intensity and frequency. However, cough resolution was significantly higher with levodropropizine than with central antitussives (47% vs. 28% respectively, p = 0.0012). The results of a logistic analysis suggest that the use of antibiotics had no significant correlation with cough improvement. Multivariate analysis showed a statistically significant difference of cough improvement with levodropropizine vs. central antitussives or no therapy, independent of antibiotic use or concomitant illnesses.

In conclusion, cough significantly disturbed children's and parents' sleep and daily activities. Levodropropizine was the antitussive drug most commonly used by the pediatricians in this study, and cough outcome in terms of resolution and improvement appeared to be better with levodropropizine than with centrally acting antitussives (cloperastine and codeine).

Further large randomized clinical trial in children should be conducted in order to confirm the efficacy of antitussive drugs used in this observational study.

## Competing interests

This study was supported by Dompé spa through an unrestricted grant.

F. De Blasio, P.V. Dicpinigaitis, B.K. Rubin and A. Zanasi are members of an International Advisory Board supported by an unrestricted educational grant from Dompé spa.

G. De Danieli and L. Lanata are employees of Dompé spa, Medical Department.

Dompé spa is a company that manufactures and commercializes levodropropizine.

## Authors' contributions

AZ carried out the conception of study and set up the task group of the authors. All authors carried out the literature review. All authors carried out the draft paper. All authors read and approved the final manuscript.
